# Supplementary material for: Are carbon emissions trading and green financial instruments synergistic? -Comprehensive quantitative research based on content analysis
Source: PLoS One. 2024 Mar 7;19(3):e0298601. doi: 10.1371/journal.pone.0298601 (PMC10919601; doi:10.1371/journal.pone.0298601)
Supplement: S1 Data — (ZIP) [file pone.0298601.s001.zip › Data/地级市Z_F工作报告.zip!/╔╜╢1⁄2/╢1⁄2╙¬╩╨/└┤╫╘╣1⁄2╓┌║┼í╛┬φ┐╦╩2╛▌═°í┐_2003.docx]

各位代表：

　　现在，我代表市人民政府向大会作工作报告，请予审议，并请各位政协委员和其他列席人员提出意见。

一、过去五年的工作

过去的五年是很不平凡的五年。五年来，在省委、省政府和市委的正确领导下，全市人民高举邓小平理论伟大旗帜，认真贯彻落实“三个代表”重要思想，艰苦奋斗，开拓创新，圆满完成了市四届人大一次会议确定的各项任务，开创了全市经济社会发展的新局面。

　　国民经济综合实力显著增强。初步统计，2002年全市国内生产总值达到540.3亿元，比1997年增长54.6%，年均增长9.1%；人均由18041元增长到30966元。其中地方国内生产总值达到248.9亿元，五年翻了一番多，年均增长15.2%。农业结构调整取得明显成效，主导产业和龙头企业不断壮大，产业化进程加快。全市经济作物产值占种植业产值的比重由53.4%提高到73.1%，畜牧、水产业产值占农业总产值的比重由28%提高到38.3%。石油工业稳步发展，2002年原油、天然气产量分别达到2671.5万吨和7.5亿立方米；油田存续企业的发展活力进一步增强。地方工业发展步伐加快，2002年地方规模以上工业增加值达到70.9亿元，五年增长2.4倍，年均增长27.9%；销售收入过亿元的企业发展到36家，增加25家。工业技术装备水平和创新能力显著提高，地方工业五年累计完成技改投入107.5亿元，全市省级以上高新技术企业发展到56家，增加36家。消费、投资不断扩大。2002年全市实现社会消费品零售总额84.4亿元，年均增长10.1%；全社会固定资产投资完成239.8亿元，其中地方完成129.8亿元，年均分别增长14.9%和34.5%。民营经济蓬勃发展。2002年全市个体工商户达到4.2万户，私营企业5305家；实现税收3.2亿元，五年增长4.6倍。财政金融实力不断壮大。2002年全市地方财政收入达到18.68亿元，五年翻了一番多，年均增长16.1%；金融机构存贷款余额分别比1997年增长141.5%和101.6%，地方贷款所占比重由37%增长到87%。

　　改革开放实现重大突破。招商引资深入实施，三年来到位外来固定资产投资164.4亿元，为经济和社会发展带来了巨大生机和活力。对外贸易连年高速增长，2002年全市进出口总值达到5.2亿美元，五年增长16.9倍，年均增长78%；其中出口2.96亿美元，五年增长24.2倍，年均增长90.7%。五年累计利用外资2.4亿美元。东营经济开发区等一批经济园区迅速崛起，成为重要的经济增长点。企业改革取得重大进展，乡及乡以上国有、集体企业改制基本完成。华泰股份成功上市，科达集团获准上市。各项配套改革取得新的进展。财政改革力度加大，推行了细化预算编制和部门预算，初步建立了财政集中支付制度。调整优化支出结构，集中财力办大事，加大了向农业农村农民、向科技教育、向基础设施建设、向弱势群体倾斜的力度。仅去年，市财政用于支持农业农村农民和救助弱势群体的资金达3.4亿元，占市级财政支出的32.6%。计划、投融资、住房等改革不断深化，整顿市场经济秩序取得阶段性成果。土地延包工作顺利完成，农村税费改革扎实推进，农民负担明显减轻。市县乡机构改革平稳进行。

　　城市和基础设施建设步伐加快。修订完善了中心城总体规划，加快了详细规划和专业规划编制步伐。五年来改造建设中心城主次干道45条，基本搭起了城市发展框架。在加快东城建设的同时，加大了西城改造力度，启动了东西城对接工程，配套建设了污水处理厂等一批市政公用设施，城市功能进一步完善。商河路商业街改造全面开工。实施了中心城碧水工程和绿化美化亮化工程，五年新增绿地面积349万平方米，人均达到9.8平方米。加强了环境综合整治，深入开展创建国家环保模范城和国家卫生城工作，理顺城市管理体制，加大管理力度，城市面貌有了很大改观。以推行土地“五统一”管理为重点，经营城市工作取得初步成效。小城镇发展步伐加快。全市城市化水平达到56.5%。大力加强基础设施建设，实施了以王庄、胜利、麻湾三大灌区节水改造为重点的水利工程建设，加强了引黄供水工作和农业综合开发，有效地改善了农业生产条件和生态环境。大搞植树造林，实施了胜利干渠绿化等工程，全市森林覆盖率由10.8%提高到14%，被评为全国平原绿化先进市。建设防潮大堤，加强黄河口综合治理，进一步增强了全市的安全防护能力。加大了城乡公路建设改造力度，五年共修建公路1614公里，公路密度达到每百平方公里56.9公里。东营机场启用通航。城乡电网改造取得明显成效，并实现了城乡居民生活用电同网同价。电话普及率达到每百人65部，移动通讯用户达到45万户。

　　油地军校结合取得丰硕成果。各方不断深化团结共识，完善运行机制，拓宽结合领域，共同发展经济和社会事业，共同建设城市和基础设施，共同维护社会稳定，开创了团结发展最好的时期。五年实施结合项目384项，总投资84.1亿元。油田帮扶乡镇工作扎实推进，实施帮扶项目184个，总投资3.2亿元。

　　社会事业全面发展。科教兴市战略深入实施，科技对经济增长的贡献率不断提高，被国家科技部确定为全国技术创新工程试点城市，并被授予全国科教兴市先进城市。不断加大教育投入，改善办学条件，农村中小学布局调整和危房改造基本完成，实现了教育信息网络“校校通”。高等教育、职业教育和成人教育发展势头良好，创办了东营职业学院和东营高级技工学校。扎实推进素质教育，教育教学质量明显提高。计划生育、国土资源管理和环境保护工作得到加强。国防教育和创建双拥模范城工作扎实开展。医药卫生、文化体育、广播电视、新闻出版、外事侨务、地名史志、文物档案、民族宗教、人民防空、无线电管理、老龄、气象等各项事业都取得了新的成绩。

　　社会主义精神文明和民主法制建设不断加强。思想道德建设不断深入，群众性精神文明创建活动广泛开展，“信用东营”建设扎实推进，社会文明程度进一步提高。普法教育和依法治市进一步加强，依法行政水平有了新的提高。自觉接受人大及其常委会的监督，支持政协和各民主党派参政议政，认真听取各方面的意见。基层民主不断扩大，村民自治和社区建设得到加强。深入开展与“法轮功”邪教组织的斗争和“严打”整治斗争，加强社会治安综合治理，及时化解各类社会矛盾，坚持不懈地抓好安全生产，维护了社会稳定。

　　发展环境不断优化。大力推进政府工作“提速”，深化行政审批制度改革，精简审批事项392项；建立行政审批服务中心和“一站式”服务大厅，提高了办事效率。全面清理行政事业性收费项目，全部取消了市级以下收费项目，每年可减轻企业和群众负担1000多万元。全面推行政务公开和服务承诺等制度，加强行政效能监察，促进了服务水平和行政效能的提高。

　　人民群众生活明显改善。城镇居民可支配收入由7496元增加到10429元，农民人均纯收入由2219元增加到3166元。就业和再就业工作成效明显，城镇登记失业率控制在2%以内。社会保障体系逐步完善，“两个确保”保持100%，城乡低保标准进一步提高，做到了应保尽保，困难群众基本生活得到保障。实施了经济适用房和廉租房建设，基本实现了居者有其屋。在全省率先基本实现了村村通柏油路、通客车、通自来水，中心城区全部通上了天然气，城乡居民生活环境进一步改善。

　　过去的五年，是凝聚各方力量，同心同德，开拓创新的五年；是经济快速发展，各项事业大踏步前进的五年；是改革开放成效最显著，人民群众得实惠最多的五年。回首五年，东营大地发生了深刻变化。国家将发展黄河三角洲高效生态经济列入“十五”计划，联合国工发组织将东营确立为国际绿色产业示范区，使东营成为我国沿海又一开放开发的热点地区；人们的思想观念有了大的转变，眼界更加开阔，呈现出朝气蓬勃、奋发向上的精神风貌；经济建设蒸蒸日上，到处是一片热火朝天、竞相发展的繁荣景象；城市面貌日新月异，广大农村欣欣向荣；发展环境大为改善，国内外客商纷至沓来。五年奋进，五年辉煌，东营正日益展现出美好的发展前景!

　　五年取得的成就，是在省委、省政府和市委的正确领导下，全市人民团结奋斗的结果，是油地军校齐心协力、携手共进，中央和省驻东营各单位积极参与、大力支持的结果。在此，我代表市人民政府向全市人民和社会各界，向所有为东营的建设和发展做出贡献的同志们、朋友们，致以崇高的敬意和衷心的感谢!

　　同时，我们也清醒地看到，经济社会发展中还存在不少困难和问题，政府工作中也存在缺点和不足。经济整体素质和竞争力还不强，尤其是科技创新能力不足，人才缺乏；城乡差距较大，农民收入不高，部分城乡居民生活还比较困难，就业和再就业压力仍然很大；精神文明和民主法制建设需要进一步加强，维护稳定的任务仍很艰巨；我们的思想观念、依法行政水平和干部队伍素质与形势发展的要求还不相适应；有的领域还存在行业不正之风。对这些问题，我们要高度重视，采取切实有效的措施认真加以解决。

　　各位代表!五年来，我们不断加深对市情和经济社会发展规律的认识，勇于探索，大胆实践，积累了丰富的经验。一是坚持解放思想，开拓创新。东营五年发展的历程，是在实践中探索、在创新中前进的历程。必须紧跟时代发展要求，立足于新的实践和新的发展，不断解放思想，勇于创新，善于创新，永葆事业发展的生机和活力。二是坚持集中精力搞建设，一心一意谋发展。发展是硬道理，是解决一切问题和矛盾的根本途径。必须把发展作为第一要务，紧紧扭住不放松，抓住一切机遇加快发展。三是坚持油地军校结合，凝聚发展合力。东营的发展，靠的是油地军校的共同奋斗。必须始终高举团结发展的旗帜，携手并肩，相互支持，促进东营的振兴和繁荣。四是坚持不断加强社会主义精神文明和民主法制建设，努力维护社会稳定。社会主义物质文明、政治文明和精神文明相辅相成，相互促进。必须大力发展先进文化，促进经济社会的协调发展；必须加强民主法制建设，推进依法治市。稳定是改革和发展的前提。必须正确处理改革、发展、稳定的关系，探索建立维护社会稳定的长效机制，在社会稳定中推进改革发展，通过改革发展促进社会稳定。五是坚持全心全意为人民谋利益，不断改进政府工作。实现、维护和发展好人民群众的根本利益是政府一切工作的出发点和落脚点。我们坚持每年为群众办十件实事，采取转移支付等措施，为基层和群众解决实际困难，受到了群众的欢迎。只要我们一心为民，就一定能够赢得群众的拥护和支持。改进政府工作是入世和市场经济发展的要求，也是人民群众的愿望。必须不断转变政府职能和行政方式，改进作风，加强廉政建设。这些经验，是全市人民智慧的结晶。坚持和发展这些经验，在实践中探索前进，我们就一定能够不断开创改革开放和现代化建设的新局面!

二、今后五年的目标任务

党的十六大确定了全面建设小康社会的奋斗目标。综观新世纪头二十年，是一个必须紧紧抓住并且可以大有作为的重要战略机遇期。我们要紧紧抓住机遇，努力加快发展，在全面建设小康社会的基础上，提前基本实现现代化，把东营建设成为经济持续发展、科技教育发达、生态环境优美、社会文明进步、人民生活富裕的地区。新世纪头二十年的奋斗目标是：国内生产总值以2000年为基数，到2008年实现第一个翻番，年均增长9%；以2002年为基数，到2010年翻一番，达到1000亿元以上，年均增长9%；到2020年再翻一番，达到2000亿元以上，年均增长8%，人均达到10万元(按现行汇率折合12000美元)以上。实现城乡一体化，东营成为现代化大城市，形成以中心城为龙头、布局合理、功能完善、环境优美、特色鲜明的城市群，成为最适宜人类居住的城市之一。社会更加民主、文明，人与环境更加和谐。城乡差别逐步缩小，人民生活殷实富足，实现人的全面发展。实现上述目标，东营必将更加繁荣富强，人民生活必将更加幸福美好，东营大地必将发生历史性巨变。美好的前景激励着我们奋斗，鼓舞着我们前进。我们的目标一定能够实现!

　　今后五年，是东营全面建设小康社会承前启后的关键阶段，是全面完成“十五”计划，制定实施“十一五”计划，实现第一个翻番的重要时期。要坚持以邓小平理论和“三个代表”重要思想为指导，全面贯彻落实党的十六大精神，解放思想，与时俱进，开拓创新，深入实施科教兴市、经济国际化、城市化和可持续发展“四大战略”。着力取得对外开放、经济体制改革、经济结构调整、区域经济社会一体化、基础设施和城市建设“五大突破”。努力实现思想解放达到新的境界、经济发展有新的跨越、科技创新和可持续发展能力有新的增强、发展环境有新的改善、精神文明和民主法制建设有新的进展、人民生活水平有新的提高“六大变化”，为实现全面建设小康社会、提前基本实现现代化的目标奠定坚实基础。

　　今后五年的目标任务是：

　　--全市国内生产总值年均增长9%，其中地方年均增长16%，经济综合实力跃上新的台阶。

　　--建立起高效生态经济的基本框架，加工制造业基地初具规模，形成以基础产业和加工制造业为支撑，高新技术产业、现代服务业快速发展的新格局，产业素质和国际竞争力显著提升。

　　--建立和完善适应发展市场经济要求、符合国际惯例的经济管理体制和运行机制，提高对外开放水平，加快经济国际化步伐。

　　--深化油地军校结合，加强城乡建设，区域经济社会一体化、城乡一体化取得明显进展，展现出现代化城市的风采。

　　--初步建成现代国民教育体系、科技和文化创新体系、全民健身和医疗卫生体系，提高全民思想道德素质、科学文化素质和健康素质，促进人的全面发展和社会进步。

　　--把经济发展的成果落实到提高人民生活水平上，使广大群众生活更加宽裕。不断提高物质生活质量，丰富精神文化生活。建立和完善社会保障体系，促进充分就业，切实保障弱势群体基本生活。

　　各位代表!东营正处在加快发展的关键时期。党中央、国务院对东营的发展寄予厚望，省委、省政府给予大力支持。经过多年来的积极探索，我们已经理清了发展思路，明确了发展方向，积累了比较丰富的经验，油地军校空前团结，全市上下精神振奋，斗志昂扬，具备了良好的发展基础和条件。只要我们善于利用一切有利因素，充分调动和发挥各方面的积极性，举全市之力，集各方之智，开拓创新，扎实苦干，就一定能够实现超常规、跨越式发展，在全面建设小康社会和现代化建设中领先一步!

三、推动经济跨越式发展

全面建设小康社会，最根本的是坚持以经济建设为中心，不断解放和发展社会生产力。要围绕“七个大力推进、一个不断提高”，集中力量突破影响全局的关键问题，推动经济的跨越式发展。

　　(一)大力推进经济国际化。当今世界，经济全球化已经成为不可阻挡的潮流。任何一个地区面对的市场都是全球化的市场，面对的竞争都是全球性的竞争，只有积极主动地融入世界经济，才能求得生存和发展。我们要顺应时代潮流，充分利用我国入世后的过渡期，全面扩大对外开放，以开放促改革、促调整、促发展。

　　坚定不移地抓好招商引资。招商引资是推进经济国际化的有效途径，是实现经济跨越式发展的战略措施，是决定东营前途和命运的关键之举。我们要抓住国际国内产业梯次转移的良好机遇，发挥我市资源丰富、区位优越、交通便利和生产要素价格低等比较优势，加大招商引资力度。继续坚持境内招商与境外招商并举，突出抓好境外招商，全民招商与专业招商并举，突出抓好专业招商，软环境建设与硬环境建设并举，突出抓好软环境建设的“三并举、三突出”工作思路，认真研究国际资本流动规律，选准重点国家和地区，积极开展专业化招商、代理招商，着力引进加工制造项目、高新技术项目、基础设施项目和资源开发等项目，努力取得更大成效。认真研究和执行好国家有关政策，不断拓宽利用外资领域，扩大利用外资规模。进一步重视和加强环境特别是软环境建设，按照与国际惯例接轨的要求，努力创造一流的服务环境、政策环境、法制环境、市场环境和人文环境，使东营成为最适合投资者创业发展的城市之一。园区是对外开放的窗口、招商引资的龙头、承接产业转移和集聚发展的有效载体。要围绕建设山东加工制造业基地，加快东营经济开发区和各县区工业园区规划建设，搞好基础设施配套，落实好优惠政策，创新运行机制，集中引进和摆布加工项目，经过五年的努力，力争将东营建设成为山东一流的加工制造业中心。

　　加快发展对外贸易。今后五年外贸进出口总值年均增长25%，其中出口年均增长25%以上。推进市场多元化，加大国际市场开拓力度。培育一批有实力的出口骨干企业，扩大外贸主体队伍。坚持以质取胜，加强国际标准认证，提高出口商品的竞争力。大力发展加工贸易，利用好国家降低关税的机遇，大力引进国外先进技术、关键设备和原材物料，发展“协作配套”项目，促进大进大出。实施“走出去”战略，鼓励支持优势企业对外投资，开展对外承包工程和劳务输出。

　　(二)大力推进区域经济社会一体化。区域经济社会一体化是油地军校结合的必然趋势，是凝聚各方力量，整合区域资源，振兴区域经济的必由之路，是生活在这一地区人民群众的共同利益所在。现在，推进区域经济社会一体化的共识已经形成，时机已经成熟。我们要倍加珍惜油地军校精诚团结的大好局面，倍加保护和调动各方面的积极性，积极推进油地军校经济的相互融合，大力促进各种经济成份的相互渗透，推进区域经济一体化、城市规划建设管理一体化、基础设施建设一体化，让东营大地上的一切劳动、知识、技术、管理和资本的活力竞相迸发，让一切创造社会财富的源泉充分涌流，汇聚成推进区域经济社会发展的强大动力。积极支持油田、石油大学和济军生产基地的改革与发展，采取有力的政策措施，支持和推动油田存续企业改制，支持油田建设工业园。深入挖掘油田和石油大学的资本、人才和技术等潜力，优化区域生产要素配置。鼓励支持各种经济成份的企业相互参股、兼并、联合，优势互补，共同发展。只要建在东营土地上的企业，不论是国有的、集体的、民营的，不论是内资的、外资的，都是东营的企业，都要一视同仁，真心实意地搞好服务，创造公平公正的竞争环境。

　　县域经济是城乡统筹发展的重要结合点，是促进区域经济协调发展的重要途径，对全市经济具有至关重要的拉动作用。要找准优势，选准突破口，走出符合自身特点的县域经济发展之路。要启动内部活力，增创发展优势，进一步打好发展基础，着力培植支柱产业和特色产业，促进就业，增加财政收入和群众收入，使县域经济跃上一个新台阶。

　　(三)大力推进经济结构调整。东营的主要矛盾是发展。要主动适应国内外市场需求的新变化，坚持在发展中调整，在调整中加快发展，提高经济的整体素质和竞争力。

　　加快工业强市进程。我市正处在工业化加速发展的时期。要坚持走新型工业化道路，突出抓投入、抓科技，到2007年确立起工业在地方经济中的主导地位。建立起多元化的投入机制，始终保持强劲的投入力度。加强技术改造，加快运用高新技术和信息化手段改造传统产业，大力提高科技含量和装备水平，把现有企业做大做强，培育和发展一批主业突出、核心竞争力强的骨干企业。建立和完善风险投资机制，深化产学研结合，加强高新技术企业孵化器、高新技术产业园区建设，鼓励各类企业建立技术中心，增强技术创新能力，加快高新技术成果的引进、开发和转化，围绕电子信息、新材料、精细化工和生物技术与制药，大力发展高新技术产业，努力推动产业产品结构的全面升级。走新型工业化道路，必须把可持续发展放在十分突出的地位，搞好生态保护和建设，合理开发利用资源，做到科技含量高、经济效益好、资源消耗低、环境污染少、人力资源优势得到充分发挥。

　　加快现代服务业发展进程。发达的服务业是现代经济的重要标志。要以健全服务功能、提高服务水平为目标，以市场化、产业化、社会化为方向，放宽准入、整合资源，改革体制、创新机制，扩大总量、提高质量，与工业化、城市化、扩大就业紧密结合，加快服务业发展。改造提升传统服务业，加快推进流通现代化，大力发展信息、旅游、现代物流、房地产、社区服务、中介服务等新兴服务业，使服务业在国民经济中的比重有较大提高。

　　加快农业和农村现代化进程。实现全面建设小康社会的宏伟目标，最繁重、最艰巨的任务在农村。我们说现在的小康还是低水平的、不全面的、发展很不平衡的小康，差距主要也在农村。没有农民的小康就没有全市人民的小康，没有农村的现代化就没有全市的现代化。我们必须更加重视农业、农村和农民问题，自觉把全面建设小康社会的工作重点放在农村。今后五年，要大力推进农业产业化、国际化、标准化、规模化，提高农业质量、效益和竞争力。加大对龙头企业的扶持力度，培育一批竞争力和辐射带动能力强的骨干龙头企业，同时积极发展农村合作经济组织，推动农业生产方式的转变，促进农业结构调整和资源开发。逐步放开粮食市场，鼓励农民按照市场经济规律大胆地进行结构调整，大力发展优质高效经济作物和畜牧、水产等特色产业。积极推进林纸一体化项目的实施。加快建立农产品质量标准体系、检验检测体系和动植物疫病防治体系，发展农业标准化生产，提高农产品品质，积极应对绿色标准和技术壁垒，加快农业国际化进程。土地直接关系农民的权益、农业的发展和农村的稳定。要坚持党在农村的基本政策，长期稳定并不断完善以家庭承包经营为基础、统分结合的双层经营体制。有条件的地方可按照依法、自愿、有偿的原则进行土地承包经营权流转，逐步发展规模经营。鼓励农民连片承包开发荒地、荒滩、荒水。进一步深化农村税费改革，减轻农民负担，保护农民利益。大力发展农村二三产业，走工业化、城镇化的路子，把农民从农业和农村中尽可能多地转移出来。继续加大对农业的支持和保护力度，加强水利、电力、公路、通讯等农村基础设施建设，加强农村生活和卫生设施建设，改善农业生产条件和农民生活环境，加强农村教育、科技、文化等社会事业建设，促进农村社会全面发展。

　　加快民营经济发展进程。民营经济已经成为加快经济发展的生力军，越来越显示出强大的生命力。要千方百计调动民间投资创业的积极性，让千军万马、千家万户各显神通。膨胀骨干优势民营企业，鼓励有基础、有实力的骨干企业做大做强。引导民营企业增加投入，推进技术进步，加强经营管理，使其尽快地上规模、上档次、上水平。进一步放宽市场准入，改善发展环境，解决融资等制约发展的关键问题，使东营成为民营经济发展最具活力的地区之一。

　　(四)大力推进经济体制改革。根据解放和发展生产力的要求，加大市场取向的改革力度，进一步提高市场化程度，增强经济发展活力。进一步深化企业改革，加快建立现代企业制度。继续推行经营管理集团控股、经营者持大股、职工自愿参股的股份制改造，完善法人治理结构，深化企业内部改革，加快经营机制转换。引导企业积极开展资本运营，优化资本结构，扩张资本规模，提高资本盈利水平。通过市场和政策引导，培育企业集团，积极推进企业上市。按照责权利相统一的原则，建立和完善国有资产管理、监督、运营体系，确保国有资产保值增值。重点培育生产要素市场，进一步健全市场体系。继续整顿和规范市场经济秩序。进一步推进计划、投融资体制和住房制度改革。继续深化价格改革，加强价格监管。支持金融体制改革，健全金融服务体系，加强金融监管，使金融更好地为经济社会发展服务。

　　(五)大力推进基础设施建设。立足于全面建设小康社会、发展高效生态经济、建设山东加工制造业基地的需要，全面规划，分步实施，加大基础设施建设力度，为经济跨越式发展创造支撑条件。加强以“两港两路”为重点的交通设施建设，形成四通八达、方便快捷的海陆空立体交通网络，努力使东营成为鲁北地区重要的交通枢纽城市。两港，一是海港，扩建东营港，逐步建成万吨级港口，开通国际国内航线，开辟东出大海的通道；二是空港，配套完善东营机场，增开国内航线，争取开通国际航线。两路，争取尽快建成环渤海高速公路和黄东大铁路。同时，加强境内路网改造和建设，实现市到县和县县之间由一级公路连接，县到乡镇和乡镇之间由二级以上公路连接，乡镇到村由三级以上公路连接，继续提高村级公路的建设和管理水平。加强能源设施建设。实施胜利电厂二期工程，做好东营港区大型火力发电项目的前期工作；积极发展公用热电项目，到2007年中心城和县城基本实现集中供热；继续搞好城乡电网改造，推进区域电网现代化。加强信息基础设施建设。整合现有网络资源，推进网络升级换代，建成结构合理、互联互通的高速宽带网，加快建设数字化东营。坚持把水利建设摆在突出位置，在今年基本完成大中型灌区节水改扩建工程的基础上，今后几年集中力量搞好支渠和田间渠的节水改造，配套完善城乡供水体系，提高水资源保障能力。强化全民节水意识，建设节水型社会。构建黄河三角洲安全防护体系。继续搞好防潮堤等防灾减灾设施建设，加强黄河口综合治理，为可持续发展创造安全稳定的环境。同时规划建设文化体育、医疗卫生、宣传舆论、生态旅游等设施。

　　(六)大力推进城市化。城市化是经济社会发展的必然趋势和客观要求。要坚持高起点规划、高质量建设、高效能管理、高效益经营，构建以中心城为龙头、县城为支撑、小城镇为依托的城镇体系。到2007年全市城市化水平达到60%以上。

　　坚持规划先行，加快总体规划的完善和专业规划、详细规划的编制，把建成区内所有土地和建设项目全部置于规划的控制之下。严格实行规划一张图、审批一支笔、建设一盘棋，维护规划的权威性和严肃性。搞好东城建设，加大西城改造力度，加快东西城对接步伐，加强城市基础设施和市政公用设施建设，完善城市功能。搞好城市设计和绿化美化亮化，丰富文化内涵，提高城市品位。推行城市管理综合执法，全面提高管理水平。加大工作力度，争取今年实现创建国家环保模范城和国家卫生城的目标。推进经营城市，搞好土地“五统一”管理，实现城市建设投资多元化。坚持以产业化发展、市场化运作、企业化管理为目标，搞好市政、绿化、环卫等公用事业改革。要继续搞好县城的规划建设和管理。按照现代城市的标准，搞好县城基础设施的规划建设，并尽快建立起与之相适应的管理体系，争取早日成为现代化水平较高的小城市。要继续加强小城镇的规划建设，积极培育各具特色的小城镇。消除不利于城镇化发展的体制和政策障碍，引导乡村企业向城镇集中，引导农村劳动力向城镇有序转移。同时，搞好村庄规划改造，开展环境优美村创建活动，推进农村生活城市化。

　　(七)大力推进公共财政框架的建立。深化财政体制改革，健全财政职能，加强财政管理，对于优化资源配置，调节收入分配，促进经济稳定增长和区域经济协调运行，促进社会各项事业全面发展，具有重大的作用。要加强财源建设，强化税收征管，做到应收尽收，保持财政收入持续稳定增长。今后五年地方财政收入平均增长10%。深化支出改革，全面推行部门预算，完善财政集中支付制度和政府采购制度。调整支出结构，大力压减一般性支出，集中财力办大事，财政资金要继续向农业农村农民倾斜，支持农业基础设施建设，支持农业结构调整，扶持农业龙头企业发展，继续减轻农民负担；向科技教育倾斜；向弱势群体和困难群众倾斜，加大对社会保障的支持力度。完善财政体制和管理制度，加强对财政收支的审计监督。牢固树立过紧日子的思想，厉行节约，勤俭办一切事业。

　　(八)不断提高人民生活水平。发展经济的根本目的是提高人民群众的生活水平和质量。要普遍增加城乡居民收入，让人民群众共享经济社会发展的成果。今后五年城镇居民可支配收入年均增长7%，农民人均纯收入年均增长6%。就业是民生之本。要大力发展有竞争力的劳动密集型产业和中小企业，大力发展就业容量大、安置成本低的服务业，大力发展个体私营经济，广开就业门路，增加就业机会。进一步完善促进就业和再就业的政策措施，改善创业环境，加强就业培训和指导，在发展经济的基础上实现充分就业，城镇登记失业率控制在3%以内。进一步完善社会保障体系，健全城镇职工养老、失业、医疗等保险制度，扩大社会保险覆盖面。完善城乡最低生活保障制度，建立农村养老保险和合作医疗保险制度。加大对困难群众和弱势群体的扶助力度，帮助他们解决生产生活中的困难。搞好扶贫开发，加快贫困人口脱贫致富步伐。实施安居工程，改善居住条件。

四、推进精神文明和民主法制建设

建设社会主义政治文明和精神文明，是全面建设小康社会的重要内容。必须坚持依法治市和以德治市相结合，努力实现社会主义物质文明、政治文明、精神文明协调发展。

　　加强精神文明建设。深入进行党的基本理论、基本路线、基本纲领和“三个代表”重要思想的宣传教育，引导广大干部群众树立中国特色社会主义的共同理想，树立正确的世界观、人生观和价值观。认真贯彻公民道德建设实施纲要，弘扬爱国主义精神，以为人民服务为核心、以集体主义为原则、以诚实守信为重点，加强社会公德、职业道德和家庭美德教育。进一步加强和改进思想政治工作，广泛开展群众性精神文明创建活动。扎实推进“信用东营”建设，着力培育政府信用、企业信用、个人信用“三大主体”，加强信用道德、信用网络、信用制度“三大体系”建设，突出抓好金融信用、中介组织信用和产品质量信用“三大突破口”，树立东营“文明立市、诚信兴业”的崭新形象。

　　全面发展各项社会事业。坚持把教育放在优先发展的战略地位，深化教育改革，优化教育结构，合理配置教育资源，提高教育质量和管理水平，推进素质教育，全面提高劳动者素质。在巩固“普九”成果的基础上，到2005年普及学前三年教育和高中阶段教育，使城乡人均受教育年限有较大提高。办好东营职业学院和高级技工学校，加强职业教育和岗位培训，发展继续教育，构建终身教育体系，建设学习型社会。加强教师队伍建设，提高教师的师德和业务水平。加大对教育的投入和对农村教育的支持，鼓励社会力量办学。深化科技体制改革，加强科技与经济的结合，完善科技服务体系，加速科技成果向现实生产力的转化。整合区域科技资源，深化油地校科技合作，鼓励发展民营科技企业和民营科研机构。普及科学知识，弘扬科学精神，提高广大干部群众的科学素养，在全社会形成崇尚科学、勇于创新、反对迷信和伪科学的良好氛围。发展社会科学，发挥其在经济社会发展中的作用。经济的竞争，归根到底是人才的竞争。要树立人才是第一资源的观念，最大限度地开发人力资源，整合区域人才资源，加强人才的培养和引进，优化人才发展环境，尊重劳动、尊重知识、尊重人才、尊重创造，努力形成广纳群贤、人尽其才的良好机制，为经济社会发展提供人才保障。大力发展先进文化和黄河口特色文化，繁荣广播影视、新闻出版事业，丰富城乡人民的精神生活。推进文化体制改革，加强文化市场管理，大力发展文化产业。积极推进医药卫生和体育事业的改革和发展，强化药品市场监管，加强卫生服务体系和医疗保健体系建设，开展全民健身运动，提高全民健康水平和竞技体育水平。认真实施三项基本国策，抓好计划生育工作，稳定低生育水平，促进优生优育，进一步搞好环境保护和国土资源管理工作。加强国防教育，抓好民兵预备役工作，大力开展双拥共建活动。进一步做好老龄、残疾人、外事、侨务、地名、史志、档案、气象、无线电管理、民族宗教、人防等工作。

　　加强民主法制建设。各级政府要自觉维护党委的领导核心地位，自觉接受人大及其常委会的监督，认真执行人大及其常委会的决议决定，认真听取人民政协、民主党派、人民团体和社会各界的意见。改革完善行政决策机制，建立健全社情民意反映制度、与群众利益密切相关的重大事项社会公示制度、社会听证制度、专家咨询制度，实行决策论证制和责任制，使行政决策更好地体现人民群众的利益和愿望。加强基层政权建设，扩大基层民主，保证人民群众依法直接行使民主权利。完善村民自治，健全民主选举、民主决策、民主管理、民主监督制度，扩大群众对公共事务的知情权、参与权、决策权和监督权。完善城市居民自治，健全并充分发挥居民委员会的作用，建设管理有序、文明祥和的新型社区。坚持和完善职工代表大会和其他形式的企业民主管理制度，推行厂务公开，维护职工的合法权益。大力推进依法治市。广泛开展法制宣传教育，增强全民的法律意识和法制观念。严格依法行政，规范行政行为，转变执法方式，做到文明执法、公正执法。加强对行政执法活动的监督，防止和纠正违法及不正当的行政行为。大力拓展和规范法律服务，积极开展法律援助。全面推进基层、行业、区域依法治理，推进各项事业依法规范、依法管理和依法运行。

　　维护社会稳定。要坚持稳定压倒一切和安全第一的方针。做好人民来信来访工作，健全经常化、制度化的矛盾排查、调处机制，正确运用经济、行政和法律手段，妥善处理人民内部矛盾。加强基层基础工作，把矛盾化解在基层。依法严厉打击各种犯罪活动，防范和惩治邪教组织的犯罪活动，坚决扫除社会丑恶现象，切实保障人民群众生命财产安全。坚持打防结合、预防为主，落实社会和油区治安综合治理的各项措施，坚持群防群治，加强和改进社会管理，建立社会治安长效机制，保持良好的社会秩序。坚持不懈地抓好安全生产，确保安全生产形势持续稳定。

五、切实加强政府自身建设

面对新形势、新任务，我们必须按照“三个代表”的要求，大力加强和改进政府工作，奋发有为，扎实苦干，肩负起历史赋予的重任。

　　转变政府职能和行政管理方式。改革政府工作中不适应市场经济发展和入世要求的方面和环节，强化政府提供公共产品、搞好公共服务、创造公平环境的职能，切实把政府职能转到经济调节、市场监管、社会管理和公共服务上来，力争用三到五年的时间，建立起规范化的新型行政管理运行机制。按照精简、统一、效能的原则和决策、执行、监督相协调的要求，继续推进政府机构改革，科学规范部门职能，合理设置机构，优化人员结构，实现机构和编制的法定化，切实解决层次过多、职能交叉、权责脱节等问题。按照政事分开原则，改革事业单位管理体制。进一步推进政府工作全面“提速”，推行政务公开，推进电子政务，降低行政成本，提高行政效率。完善行政效能监察机制，建立科学合理的行政效能监察评估体系，强化行政效能监察工作，促进行政管理水平的提高。

　　大力提高政府工作人员的整体素质。各级政府要更好地为工作人员创造学习和实践锻炼的条件，加强教育培训。政府工作人员要勤于学习，善于学习，孜孜不倦地汲取新的理论、新的知识，不断丰富头脑，开阔视野，积累经验，增长本领，同时要大胆实践，肯于思考，努力成为本职工作的行家里手。政府工作人员特别是领导干部要讲政治、讲大局，不断提高科学判断形势的能力、驾驭市场经济的能力、应对复杂局面的能力、依法行政的能力和总揽全局的能力。要公正用人，选拔任用优秀人才，努力建设一支高素质的公务员队伍。

　　切实转变作风。全面建设小康社会是全新的实践。必须始终坚持解放思想，实事求是，与时俱进，积极探索加快发展的新途径、新办法，勇于创新，敢为人先，不断开创各项工作的新局面。加强调查研究，及时解决经济社会发展中出现的矛盾和问题。进一步完善责任制和督查考核机制，确保各项任务落到实处。领导干部要求真务实，埋头苦干，做奋发有为的领导者。

　　加强廉政建设。认真落实廉政建设责任制，进一步抓好领导干部廉洁自律，严肃查处腐败案件，切实纠正部门和行业不正之风。坚持标本兼治、综合治理，逐步加大治本的力度，加强教育，创新体制，健全制度，强化监督，把反腐败寓于各项重要政策措施之中，从源头上预防和解决腐败问题。各级领导干部必须以身作则，加强世界观改造，继承和发扬艰苦奋斗的优良传统，始终做到清正廉洁，自觉与各种腐败现象作坚决斗争。对任何腐败分子都必须彻底查处，严惩不贷。

　　全面建设小康社会，必须充分调动和激发人民群众的积极性和创造力，必须紧紧依靠各方面的团结。我们要坚持执政为民，深怀爱民之心，体察民情民意，关心群众疾苦，扎扎实实为群众办实事、办好事。要紧紧团结群众，依靠群众，集中群众的智慧，带领群众创造幸福生活和美好未来。要加强上上下下、方方面面的团结，凝聚起加快发展的强大合力。全面建设小康社会，加快东营现代化建设，任重道远，在前进的道路上会遇到各种可以预料和难以预料的困难和风险，但只要我们各级领导始终保持奋发有为的精神状态和昂扬锐气，只要有了人民群众的信赖和支持，只要有了全市上下的精诚团结，我们就一定能够冲破一切艰难险阻，无往而不胜!

　　各位代表，今年是本届政府任期的第一年，也是深入贯彻党的十六大精神、全面建设小康社会的第一年。我们要扎扎实实抓好各项工作，开好局、起好步。今年全市经济和社会发展的主要预期目标是：国内生产总值增长9%，其中一二三产业分别增长6%、8%和14%；地方国内生产总值增长16%；地方财政收入增长10%；全社会固定资产投资增长17%，其中地方增长35%；社会消费品零售总额增长11%；外贸出口增长25%，吸收外商直接投资增长30%；城镇居民可支配收入增长8%，农民人均纯收入增长6%；城镇登记失业率控制在2%以内；人口自然增长率控制在9‰以内。我们要集中精力抓好经济建设，确保完成今年的各项目标任务，并力求发展得更快、更好一些。大力推进大开放、大招商，加快发展加工制造业；深化各项改革，增强发展活力；搞好经济结构调整，加大投入，抓好企业技术改造、信息化改造和技术创新，推进农业产业化，加快发展现代服务业，放手发展民营经济，发展壮大县域经济；加大城市和基础设施建设力度；大力增收节支，坚决反对铺张浪费、讲排场比阔气的奢侈之风。进一步推进社会主义精神文明建设和民主法制建设，大力发展各项社会事业，推动社会全面进步。围绕构建农民健康保障体系，实施老年幸福工程和无障碍设施建设，解决农村中小学生“吃住”问题，继续搞好市直机关经济适用房建设并启动企业经济适用房建设，建设百座利民桥，改造中心城居住小区及逐步实施居住小区分质供水，建设和改造中心城公厕及机关、学校和企事业单位内部厕所，改造城乡首集市场，规划建设黄河口旅游南线及市区部分汽车燃气改造等方面，为市民办好十件实事。围绕东营港扩建，渔港建设，国家环保模范城市和卫生城市创建，大中型灌区节水改造，10万亩造纸林示范项目，中心城水域治理，以西城为主的城市道路建设和改造，城乡公路建设改造和黄河堤防道路硬化，“数字化东营”城域网建设及百姓平安工程等方面，建设好十大工程。

　　各位代表!宏伟的蓝图已经绘就，东营的未来灿烂美好。让我们高举邓小平理论伟大旗帜，全面贯彻“三个代表”重要思想，在市委的正确领导下，万众一心，奋发图强，为全面建设小康社会，加快东营现代化建设而努力奋斗!
